# Evolution of antimicrobial resistance in *E. coli* biofilm treated with high doses of ciprofloxacin

**DOI:** 10.3389/fmicb.2023.1246895

**Published:** 2023-09-05

**Authors:** Live L. Nesse, Ane Mohr Osland, Basma Asal, Solveig Sølverød Mo

**Affiliations:** ^1^Department of Food Safety and Animal Health Research, Norwegian Veterinary Institute, Ås Municipality, Norway; ^2^Department of Microbiology, Norwegian Veterinary Institute, Ås Municipality, Norway; ^3^Department of Bacteriology, Norwegian Veterinary Institute, Ås Municipality, Norway

**Keywords:** antimicrobial resistance, biofilm, evolution, ciprofloxacin, *E. coli*

## Abstract

The evolution of antimicrobial resistance (AMR) has mainly been studied in planktonic bacteria exposed to sub-inhibitory antimicrobial (AM) concentrations. However, in a number of infections that are treated with AMs the bacteria are located in biofilms where they tolerate high doses of AM. In the present study, we continuously exposed biofilm residing *E. coli* at body temperature to high ciprofloxacin (CIP) concentrations increasing from 4 to 130 times the minimal inhibitory concentration (MIC), i.e., from 0.06 to 2.0 mg/L. After 1 week, the biofilms were full of CIP resistant bacteria. The evolutionary trajectory observed was the same as described in the literature for planktonic bacteria, i.e., starting with a single mutation in the target gene *gyrA* followed by mutations in *parC*, *gyrB*, and *parE*, as well as in genes for regulation of multidrug efflux pump systems and outer membrane porins. Strains with higher numbers of these mutations also displayed higher MIC values. Furthermore, the evolution of CIP resistance was more rapid, and resulted in strains with higher MIC values, when the bacteria were biofilm residing than when they were in a planktonic suspension. These results may indicate that extensive clinical AM treatment of biofilm-residing bacteria may not only fail to eradicate the infection but also pose an increased risk of AMR development.

## Introduction

Antimicrobial resistance (AMR) is a threat to global human and animal health, food safety and security, and development, and therefore one of the most important challenges facing society today (World Health Organization [WHO], 2020). Acquired AMR in bacteria is a property that is evolved through mutations in chromosomal genes, or by gain of resistance gene(s). A common approach for studying evolution of AMR is to use adaptive evolution to identify resistance-conferring mutations. Mutation rate is a key determinant of evolution. Variability in this rate has been shown in different scenarios to play a key role in evolutionary adaptation, and resistance evolution under stress caused by selective pressure from antimicrobials (AMs) (Engelhardt and Shakhnovich, 2019). Numerous AMR evolution studies have been performed on planktonic bacteria (Nichol et al., 2019; Rosenkilde et al., 2019). In contrast, and despite the predominance of biofilm growth in nature, very few evolution experiments have been performed on biofilm populations (Ahmed et al., 2018; France et al., 2019; Santos-Lopez et al., 2019; Trampari et al., 2021; Usui et al., 2023). Bacterial biofilms consist of bacteria attached to a surface and/or each other and embedded in a self-produced matrix, and have been shown to be present and part of the pathogenesis in bacterial infections in most body system of humans as well as animals (Vestby et al., 2020; Nesse et al., 2023). Bacteria in biofilms show a temporary phenotypic tolerance to antimicrobials (Olsen, 2015; Bowler, 2018). This tolerance is multi-factorial, and it is attributed to restricted penetration of the antibiotics, restricted growth at low-oxygen tension, expression of biofilm-specific genes and the presence of persister cells (Ciofu et al., 2017). Consequently, bacteria in biofilms tolerate higher doses of AMs than planktonic bacteria.

Ciprofloxacin (CIP) is a fluoroquinolone, i.e., a group of AMs considered to be critically important for human medicine where they are used for treatment of a variety of infections (World Health Organization [WHO], 2017; El-Attar et al., 2022). Fluoroquinolones cause bactericidal DNA damage by forming a topoisomerase-quinolone-DNA ternary complex, which leads to double-stranded breaks in DNA and blocking of DNA replication (Dalhoff, 2012). The DNA damage triggers the SOS-response, which induces DNA repair as well as mutagenesis (McKenzie et al., 2000). Development of bacterial resistance against CIP is a growing concern. As many of the diseases that are treated with CIP may be biofilm associated, the role of biofilm in the evolution of CIP resistance is of special interest. Mutations in the gyrase and topoisomerase genes are generally the primary mechanisms of such resistance (Hamed et al., 2018). However, the few studies that have addressed this question, have observed none or few of these target specific mechanisms in biofilm evolved resistance (Ahmed et al., 2018; France et al., 2019; Santos-Lopez et al., 2019; Trampari et al., 2021).

**FIGURE 1:**
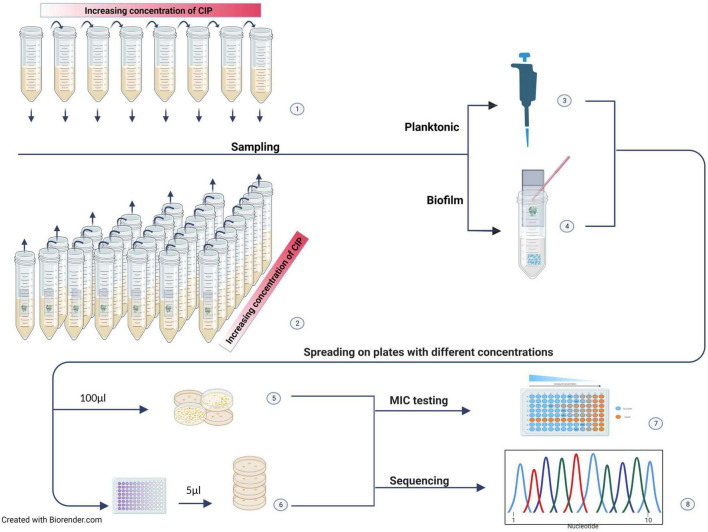
Experimental design. (1) The planktonic suspension was incubated in increasing CIP concentrations starting with 0.5 × MIC. Samples were taken after each incubation period (2–3 days). (2) Seven parallel biofilms on glass slides were separately incubated in increasing CIP concentrations starting with 4 × MIC. One biofilm was sampled after each incubation period. (3) The planktonic sample was used directly for further investigations. (4) The biofilm was scraped off the glass slide, disrupted, and the cells suspended in sterile saline. (5) For enumeration, the suspensions were serially diluted, and (6) spread on LB agar plates without CIP and selective agar plates with different CIP concentrations. Selected colonies were subjected to MIC testing (7) and whole genome sequencing (WGS) (8).

In the present study, we chose to expose biofilm residing *E. coli* to lethal concentrations of CIP continuously for 2 weeks, as in a clinical treatment plan. The hypothesis was that treatment of biofilms with CIP concentrations above their MIC value might not only fail to eradicate the bacteria, but also stimulate them to evolve a high-level resistance against CIP. We also wanted to see if the evolutionary trajection of resistance under such conditions differed from what would be observed in planktonic cultures. To investigate this, we used the experimental design described in Figure 1.

## Materials and methods

### Bacterial strain and media

The *E. coli* strain used (2014-01-6355) was originally collected from broiler chicken cecum as part of the Norwegian monitoring program on AMR in the veterinary sector (NORM-VET) (Simonsen and Urdahl, 2014). The isolate was susceptible to CIP with a minimum inhibitory concentration (MIC) ≤0.015 mg/L. Furthermore, it produced biofilm with both curli fimbriae and cellulose in the matrix, i.e., the RDAR morphotype in the Congo red agar assay (Nesse et al., 2021). The strain was stored at −80°C in brain heart infusion broth (BHI; Difco, BD, Franklin Lakes, NJ, USA) supplemented with 15% glycerol (Merck KGaA, Darmstadt, Germany) and recovered on blood agar (cattle blood) at 37.0 ± 1.0°C overnight. Lysogeny broth (LB; Merck) was used for overnight culture and in all experiments on planktonic growth. LB without NaCl (LB^wo^/NaCl; Bacto-tryptone 10 g/liter, yeast extract 5 g/liter) was used for the biofilm experiments. The antimicrobial CIP (Ciprofloxacin hydrochloride; Bayer; Germany) was added to the media to the desired concentrations.

### Experimental design for the evolution of antimicrobial resistance

An overview of the experimental design is given in Figure 1. Evolution was studied under increasing concentrations of CIP at 37.0 ± 1.0°C. Based on the results from preliminary studies, the experiment was performed twice. In each experiment, a start suspension was made by mixing blood agar colonies in LB^wo^/NaCl to a concentration corresponding to the 1.0 ± 0.1 McFarland standard. The same start suspension was used to produce both biofilms and planktonic test suspensions.

#### Evolution in biofilm residing bacteria

Parallel biofilms were produced on 16 glass slides by adding 500 μL start suspension to each of 16 centrifuge tubes (50 mL, Sarstedt AG & Co KG, Nürnbrecht, Germany) containing 10 mL LB^wo^/NaCl and an autoclaved microscope slide (76 by 26 mm; Menzel GmbH + CoKG, Braunschweig, Germany). The tubes were incubated at 37.0 ± 1.0°C for 48.

After incubation, biofilms on two randomly selected slides were harvested (sampling 0), serially diluted, and spread on LB agar plates without CP for enumeration of the total number of colony forming units (cfu), and on selective LB agar plates with 0.25, 0.5 or 1 mg/L CIP for enumeration of CIP resistant cfu (For details, see section “Sampling”).

The rest of the glass slides were randomly allocated to receive CIP (BIO CIP) or control (BIO CTR) treatment, and accordingly each placed in a separate test tube with the designated medium for incubation, i.e., 10 ml LB^wo^/NaCl with 0.06 mg/L CIP or without CIP, respectively. The initial concentration of CIP was four times the strain’s MIC value. The tubes were incubated in 37.0 ± 1.0°C.

Every second day (third day at weekends), the biofilm of one random slide from each treatment group was harvested and investigated as described for sampling 0. The rest of the slides were moved to tubes with fresh medium with or without CIP, according to their allocated treatment. The CIP concentration was doubled every time the slides were moved (Table 1).

**TABLE 1:** Description of treatment alternatives.

Short name	State of the bacteria	Treatment
BIO CTR	Biofilm	No antimicrobials, i.e., control
BIO CIP	Biofilm	CIP concentration in each incubation period: 0.06, 0.12, 0.25, 0.5, 1.0, 2.0, and 2.0 mg/L
PLANK CTR	Planktonic	No antimicrobials, i.e., control
PLANK CIP	Planktonic	CIP concentration in each incubation period: 0.0075, 0.015, 0.03, 0.06, 0.12, 0.25, and 0.25 mg/L

Selected colonies from relevant samplings were subjected to MIC testing and whole genome sequencing (WGS).

#### Evolution in planktonic bacteria

First, 500 μL of the start suspension were added to a 50 mL centrifuge tube containing 10 mL LB, and incubated at 37.0 ± 1.0°C for 48 h.

After incubation, a sample of the suspension (sampling 0), was serially diluted and spread on both LB agar plates without CIP for enumeration of the total number of colony forming units (cfu), and on selective LB agar plates with 0.25, 0.5, or 1 mg/L CIP for enumeration of CIP resistant cfu (For details, see section “Sampling”).

Thereafter, 500 μL of the planktonic suspension were placed in each of two tubes with 10 mL fresh LB, one with CIP 0.0075 mg/L (PLANK CIP) and the other without CIP (PLANK CTR). The initial concentration of CIP was half the strain’s MIC value. The tubes were incubated in 37.0 ± 1.0°C.

Every second day (third day at weekends), one sample from each incubated tube was investigated as described for sampling 0. Furthermore, 500 μL of the planktonic suspension from the PLANK CIP tube were moved to a new tube with 10 mL fresh LB with CIP. The CIP concentration was doubled every time the slides were moved (Table 1). Likewise, 500 μL of the planktonic suspension from the PLANK CTR tube were moved to a new tube with 10 mL fresh LB without CIP.

Selected colonies from relevant samplings were subjected to MIC testing and WGS.

### Sampling

During incubation, biofilm was formed on both sides of the microscope slides at the liquid-air interface. When a biofilm was harvested, the glass slide was washed three times in sterile saline to remove loosely adhered bacteria. Thereafter, the biofilm was removed by scraping with a sterile cell scraper (BD Falcon, Bedford, MA, USA) into a sterile reagent tube containing 5 mL sterile saline and 20–30 glass beads (3 mm; Assistent, Glaswarenfabrik Karl Hecht GmbH & Co KG, Bavaria, Germany). The tube was vortexed at 2000 rpm for 1 min before the suspension was used for enumeration of total cfu by serially diluting 200 μL suspension in sterile saline and placing 5 μL dilution on agar plates. In addition, 100 μL undiluted suspension were spread directly on agar plates. Same procedure was used for enumerating total cfu in the planktonic samples, using the test suspensions of these tubes without further preparation. LB agar plates without CIP were used for enumeration of total cfu, whereas LB agar plates with three different concentrations of CIP, namely 0.25 mg/L (CIP low), 0.5 mg/L (CIP medium), and 1.0 mg/L (CIP high), were used to calculate the number of CIP resistant bacteria. The results were expressed as log_10_ cfu in the biofilm or in the 10 mL planktonic test suspension. The limit of detection was 1.7 log_10_ cfu.

### Selection of colonies for further testing

One colony from each of the samplings 0, 1, 2, 4, 6, and 7 was randomly picked from the LB agar plates without CIP used for the enumeration of BIO CTR (*n* = 6) and PLANK CTR (*n* = 6) in the experiment 2. Colonies from BIO CIP (*n* = 8) and PLANK CIP (*n* = 16) were selected from agar plates with different CIP concentrations at relevant samplings at both experiments. All selected colonies (*n* = 37) were subjected to MIC testing and whole genome sequencing together with the wildtype strain.

### Antimicrobial susceptibility testing (MIC testing)

Minimal inhibitory concentrations were determined by broth microdilution with Sensititre™ gram-negative antimicrobial susceptibility plates (EUVSEC, Sensititre, TREK Diagnostic LTD, Thermo Scientific USA), according to the manufacturer’s instructions. The CIP MIC values were classified according to the clinical breakpoints given in European Committee on Antimicrobial Susceptibility Testing Version 11.0, 2021^1^ as resistant: MIC > 0.5 mg/L, intermediate: MIC = 0.5 mg/L, or susceptible: MIC ≤ 0.25 mg/L.

### DNA extraction and whole genome sequencing (WGS)

DNA from each isolate was extracted using the QIAamp^®^ DNA Mini Kit (Qiagen) according to the manufacturer’s protocol, with some minor modifications. The optional RNase A step was included, and 100 μL 10 mM Tris, pH 8, was used as elution buffer. The DNA extracts purity was determined using MySpec (VWR^®^) and DNA concentrations using Tecan Spark Fluorometer (Tecan Trading AG, Switzerland) with the Qubit broad range kit. The DNA was prepared using the Nextera™ DNA Flex library preparation kit (Illumina) and sequenced on Illumina MiSeq resulting in 300 bp paired-end reads.

### Bioinformatic analyses

All reads were quality checked using fastQC^2^ and multiQC (Ewels et al., 2016). The reads from the wildtype isolate were trimmed using Trimgalore 2019^3^ and assembled using Unicycler (Wick et al., 2017) in normal mode. Thereafter, Prokka (Seemann, 2014) was used to annotate the genome. In order to determine changes in the genome (i.e., SNPs and indels) of isolates grown in biofilm and planktonic suspension with and without CIP, we used Snippy^4^ with the annotated wildtype genome as reference.

### Statistical analysis

A two-tailed Student’s *t*-test was used for comparisons among populations. The differences were considered statistically significant when the *p*-value was ≤ 0.05.

## Results

### Total and resistant log_10_ cfu during evolution in biofilm

**FIGURE 2:**
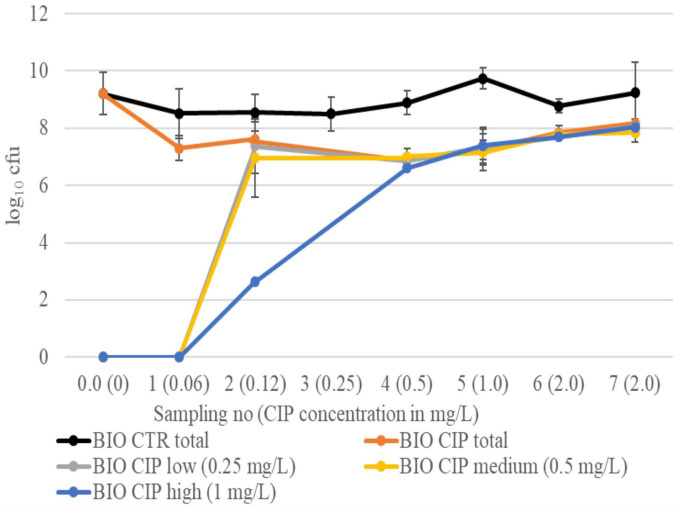
Total log_10_ cfu in BIO CIP and BIO CTR, as well as log_10_ cfu growing on selective plates with low (0.25 mg/L), medium (0.5 mg/L), and high (1.0 mg/L) CIP concentrations, at different sampling points. Results are given as means of experiments 1 and 2. Bars represent standard deviations. Results from sampling 3 in BIO CIP are missing in both experiments.

Throughout the experiments, the total log_10_ cfu in the biofilms of BIO CIP was lower than that of BIO CTR (mean difference ± SD = 1.44 ± 0.65, *p* = 0.01). The BIO CIP treated bacteria displayed the same resistance evolution pattern in both experiments (Figure 2). Bacteria growing on CIP low and CIP medium agar plates were first observed at sampling 2, and the mean log_10_ cfu on these plates were not statistically different from the total mean log_10_ cfu in the biofilm throughout the rest of the experiments (*p* = 0.78 and 0.61, respectively). At the same sampling, low numbers of cfu growing on CIP high agar plates were also observed. Unfortunately, results from sampling 3 were missing from both experiments. Consequently, it is not known whether these numbers reached the level of the total mean log_10_ cfu at sampling 3 or at sampling 4 where this was first observed.

### Total and resistant log_10_ cfu during evolution in planktonic suspension

**FIGURE 3:**
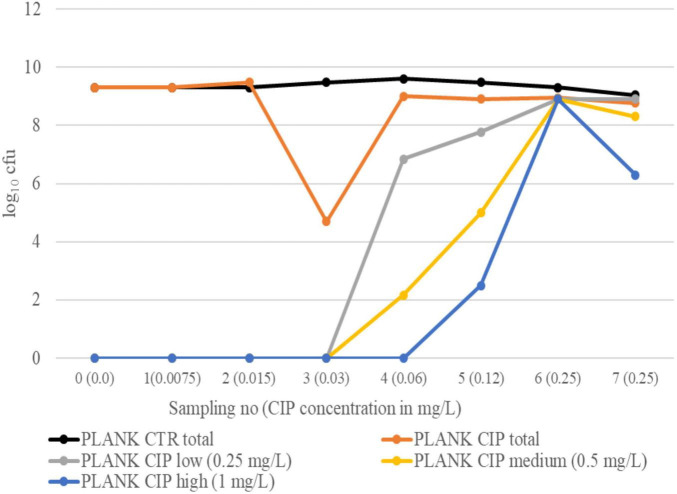
Experiment 1. Total log_10_ cfu in PLANK CIP and PLANK CTR, as well as log_10_ cfu growing on selective plates with low (0.25 mg/L), medium (0.5 mg/L), and high (1.0 mg/L) CIP concentrations, at different sampling points.

**FIGURE 4:**
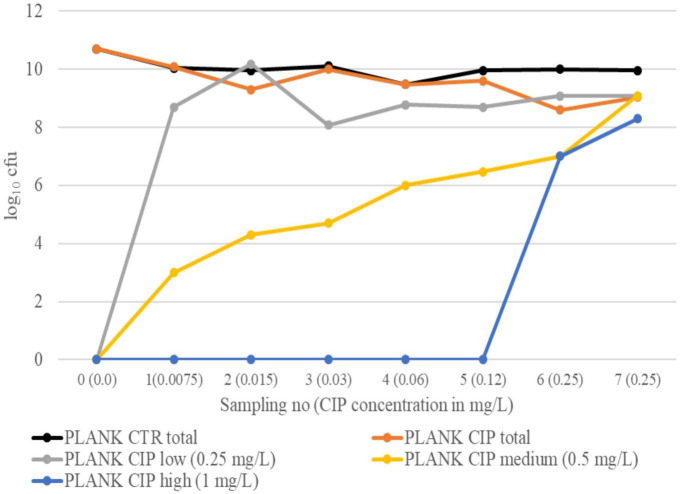
Experiment 2. Total log_10_ cfu in PLANK CIP and PLANK CTR, as well as log_10_ cfu growing on selective plates with low (0.25 mg/L), medium (0.5 mg/L), and high (1.0 mg/L) CIP concentrations, at different sampling points.

Interestingly, the pattern of resistance development in CIP-exposed planktonic bacteria differed in the two experiments. In experiment 1, a sharp drop in total log_10_ cfu of PLANK CIP was observed at sampling 3, and bacteria growing on CIP agar plates were not observed until sampling 4 (CIP low and CIP medium) and sampling 5 (CIP high) (Figure 3). Although the total log_10_ cfu of PLANK CIP increased at sampling 4, it remained lower than the total log_10_ cfu of PLANK CTR for the rest of the experiment (*p* = 0.006). In experiment 2, bacteria growing on CIP low and CIP medium agar plates were observed already at sampling 1, whereas bacteria growing on CIP high plates were not seen before sampling 6 (Figure 4). Also in this experiment, the total log_10_ cfu of PLANK CIP total was lower than PLANK CTR in the last three samplings (*p* = 0.05).

### MIC testing

The wildtype strain had a CIP MIC value of ≤0.015 mg/L. All isolates from the controls displayed CIP MIC values in the range of ≤0.015–0.03 mg/L, i.e., the same as the wildtype strain within the accepted error range of one dilution step (European Food Safety Authority [EFSA], Aerts et al., 2019; Supplementary Tables 1, 2). The BIO CIP isolates had CIP MIC values ranging from 1 to >8 mg/L (Table 2), whereas the PLANK CIP isolates displayed values from 0.5 to 2 mg/L (Tables 3, 4). In addition, a number of the BIO CIP and PLANK CIP isolates, but none of the control isolates, had increased MIC values for chloramphenicol (CHL) and tetracycline (TET).

**TABLE 2:** Results from MIC testing and WGS of selected isolates from the two BIO CIP experiments.

	BIO CIP experiment 1					BIO CIP experiment 2			
	Sampling no.	2	2	4	7	2	2	6	7
	Strain no.	30	31	32	33	34	35	36	37
	MIC CIP	1	1	2	8	1	2	4	>8
	MIC CHL	≤8	16	32	32	32	32	≤8	16
	MIC TET	≤2	4	8	8	8	8	≤2	8
	Total no. of mutations	6	5	7	10	5	4	6	8
Topoisomerases	*gyrA*	Ser83Leu	Ser83Leu	Asp87Asn	Asp87Gly	Asp87Gly	Asp87Gly	Asp87Asn	Ser83Leu
	*gyrB*				Ser464Tyr			Ser464Tyr	Glu466Asp
	*parC*	Ser80Arg	Ser80Arg					Ser80Arg	Ser80Arg
	*pare*				Ser458Pro				
Multidrug efflux pump systems	*marR*			Gly104Cys		Asp67Tyr	Asp67Tyr		Frameshift
	*acrR 1*			Deletion	Frameshift	Gln78Lys	Gln78Lys		Arg13Leu
	*rob 1*				Gln194Leu				
Porin regulation	*ompF*								Stop gained
Other genes	*clpX*				Leu348Gln				
	*dnaB 2*			Phe61Leu					
	*hemX*			Deletion					
	*Hpt*				Arg78Ser				
	*mngB*					Leu617Leu	Leu617Leu		
	*mnmE*	Frameshift							
	*mnmG*			Frameshift					
	*nrdA*							Gly163Gly	
	*pgm*								Gly494Val
	*prlF*					Frameshift			
	*prmC*								Frameshift
	*rfbA*				Frameshift				
	*rne*							Frameshift	
	*sspA*			frameshift					

MIC values are given in mg/L. CHL, chloramphenicol; TET, tetracycline. Only mutations in annotated genes are shown. For details on all mutations, see Supplementary File 3.

**TABLE 3:** Results from MIC testing and WGS of selected isolates from PLANK CIP experiment 1.

		PLANK CIP experiment 1				
	Sampling no.	4	5	5	6	7
	Strain no.	25	26	27	28	29
	MIC CIP	0.5	0.5	1	1	1
	MIC CHL	32	32	16	16	16
	MIC TET	4	8	8	8	4
	Total no. of mutations	4	6	10	6	7
Topoisomerases	*gyrA*				Asp87Gly	Asp87Gly
	*gyrB*	Ser464Tyr	Ser464Tyr	Ser464-Glu466 deletion	Ser464Tyr	Ser464Tyr
Multidrug efflux pump systems	*marR*	Frameshift		Frameshift		
	*acrR1*		Trp63Arg	Frameshift		
	*soxR*		Arg51His		Arg51His	Arg51His
Other genes	*chuR2*			Frameshift		
	*dhaM*					Stop gained
	*fabR*				Met93Ile	
	*fimH2*			Deletion		
	*focB*		Thr108Ala			
	*mnmA*			Frameshift		
	*rfbC*	Frameshift	Frameshift		Frameshift	Frameshift
	*rfbD*			Insertion		
	*rpoA*					Thr27Pro
	*ruvA*			Insertion		
	*sspA*			Frameshift		

MIC values are given in mg/L. CHL, chloramphenicol; TET, tetracycline. Only mutations in annotated genes are shown. For details on all mutations, see Supplementary File 5.

**TABLE 4:** Results from MIC testing and WGS of selected isolates from PLANK CIP experiment 2.

	PLANK CIP experiment 2											
	Sampling no.	1	1	2	2	3	3	4	5	5	6	7
	Strain no.	14	15	16	17	18	19	20	21	22	23	24
	MIC CIP	0.5	1	2	1	0.5	2	0.5	0.5	1	2	2
	MIC CHL	≤8	16	16	≤8	≤8	≤8	≤8	≤8	16	64	16
	MIC TET	≤2	4	4	2	4	4	≤2	≤2	4	8	4
	Total no. of mutations	3	3	2	1	1	2	3	7	5	6	6
Topoiso-merases	*gyrA*	Ser83Leu	Ser83Leu	Ser83Leu	Ser83Leu	Ser83Leu	Ser83Leu	Ser83Leu	Ser83Leu	Ser83Leu	Ser83Leu	Ser83Leu
Multidrug efflux pump systems	*marR*		Arg94His	Frameshift			Arg94His				Frameshift	
	*soxR*									Arg127Pro		Frameshift
Other genes	*rne*							Frameshift				
	*rfbC*							Frameshift				
	*fimH2*									Deletion	Deletion	
	*aer*								Ala64Val			Ala64Val
	*fldA*		Gln91Leu									
	*hflD*	Glu212Ala										
	*mlaB*	Arg49Arg										
	*mnmE*								Asp253Gly			
	*mog*								Frameshift			
	*purL*										Arg910Arg	
	*yqjA*											Gly176Cys

MIC values are given in mg/L. CHL, chloramphenicol; TET, tetracycline. Only mutations in annotated genes are shown. For details on all mutations, see Supplementary File 5.

### Genetic studies

The BIO CIP isolates from the two experiments displayed from 4 to 10 mutations as compared to the wildtype strain (Table 2), whereas 1 to 5 mutations was seen in the BIO CTR isolates (Supplementary Table 1). Mutations in the QRDR of *gyrA* were observed in all BIO CIP isolates. Several isolates also had mutations in the relevant regions of *gyrB*, *parC*, and *parE*, as well as in regulator genes of multidrug efflux systems and outer membrane porin F. The isolates with the highest CIP MIC values had the highest number of mutations in these groups of genes. Other mutations appeared randomly distributed. Mutations in the BIO CTR isolates also seemed randomly distributed except for mutations at different positions in the *fimH2* gene of four isolates, and identical mutations in the *pgm* gene of three isolates. The *pgm* mutation was also observed in one BIO CIP isolate, whereas none of the BIO CIP isolates carried any of the *fimH2* mutations.

The two planktonic experiments, on the other hand, displayed different mutation patterns in their PLANK CIP isolates. In experiment 1 (Table 3), four to ten mutations per isolate were observed. All isolates had mutations in *gyrB*, as well as in regulator genes of multidrug efflux systems. In the isolates from the two last samplings, mutations in the QRDR of *gyrA* were also present. In addition, four of the five isolates investigated displayed an identical frameshift variant in the gene *rfbC*, whereas the fifth isolate had a conservative inframe insertion in *rfbD*. In contrast, all isolates in experiment 2 (Table 4) had an identical mutation in the QRDR of *gyrA*. Half of the isolates also displayed a mutation in either *marR* or *soxR*. The total number of mutations per isolate in experiment 2, was one to seven. A total of zero to six mutations per isolate was observed in the PLANK CTR isolates (Supplementary Table 2). Three isolates displayed identical mutations in the genes *fimH2*, *flu1*, and *yohF*, whereas the rest of the mutations appeared randomly distributed. The *fimH2* mutation was different from those observed in BIO CTR isolates.

## Discussion

The present study demonstrated rapid evolution of CIP resistance in biofilm-residing *E. coli* subjected to prolonged treatment with high, increasing concentrations of CIP, where the initial concentration corresponded to 4 × MIC of the wildtype strain. Already after 1 week, the biofilms were dominated by CIP resistant *E. coli*. To the best of our knowledge, this is the first time evolution of AMR in bacteria in biofilm continuously treated with high AM concentrations has been demonstrated. Our results indicate that prolonged AM treatment may not only fail to eradicate chronic bacterial infections associated with the presence of biofilms, but also result in development of high level AMR.

Strikingly, the evolutionary trajectory of AMR in the biofilms was similar in both our experiments, even though six separately developed biofilms were studied in each experiment. All CIP resistant isolates investigated displayed mutations in the QRDR of *gyrA*, and 62.5% had additional mutations in *gyrB* and/or *parC*/*parE*. Furthermore, 62.5% of the isolates had mutations in genes associated to multidrug efflux pumps and outer membrane porin regulation. This probably also contributed to the increased MIC values for CHL and TET, in addition to CIP. Higher numbers of CIP resistance-conferring mutations were correlated with higher CIP MIC values. On selective plates, colonies were first observed on those with the low and medium CIP concentrations, followed by increasing cfu numbers on the plates with high concentration. All these observations fit a stepwise evolution model as described in literature. DNA gyrase is the primary target of fluoroquinolones in *E. coli*, but if *gyrA* is mutated, topoisomerase IV becomes a target (Redgrave et al., 2014). Accordingly, the primary event of evolving resistance in planktonic cultures is believed to be a single mutation in *gyrA* followed by mutations in *parC* and the *mar* operon, where each new mutation increases the level of resistance (Huseby et al., 2017). This is also what was seen in the biofilm residing bacteria in our study. The observed positions substituted in GyrA (Ser-83 or Asp-87) and ParC (Ser-80) are reported to be the most commonly found in CIP resistant *E. coli* (Jacoby, 2005). The position mutated in GyrB interacts with the quinolone molecule when this is bound to the QRDR of GyrA (Bhatnagar and Wong, 2019). Mutations at the same position in ParE as in our experiment have also been related to increased MIC-values (Sorlozano et al., 2007). Although the biofilm lifestyle itself has been reported to induce AMR conferring mutations (Nesse and Simm, 2018; France et al., 2019), no growth was observed on selective CIP plates in our BIO CTR experiments, nor did any of the isolates investigated have mutations related to quinolone resistance. This confirms that the evolved resistance in BIO CIP was due to the CIP exposure.

Interestingly, our results differed from earlier studies on evolution of CIP resistance in biofilm with *Pseudomonas aeruginosa* (Ahmed et al., 2018, 2020) and *Acinetobacter baumannii* (Penesyan et al., 2019; Santos-Lopez et al., 2019). In these studies, the biofilm cells only acquired mutations in regulators of efflux pumps, and not in the topoisomerase target genes. Accordingly, their CIP MIC values were lower than those of planktonic evolved cells. Likewise, a CIP resistance evolution experiment in *Salmonella enterica* serovar Typhimurium biofilms, displayed in a wide variety of mutations resulting in porin loss, efflux changes and in some cases target site changes, indicating multiple paths of evolution and resistance (Trampari et al., 2021). However, the experimental design in all these studies deviated from our study at two major points. Firstly, their biofilms were exposed to sub-inhibitory CIP concentrations, either throughout the experiment, or initially with subsequent increasing concentrations. Secondly, the lifespan of the exposed biofilms were short, i.e., 1 to 3 days. One study had a total exposure time of 3 days (Penesyan et al., 2019), whereas the others investigated short term biofilms repeatedly propagated from the previous biofilms. Our results show that the biofilm residing bacteria can mount a target based evolutionary trajectory and high CIP MICs, if exposed to high CIP concentrations and given sufficient time.

Ciprofloxacin is known to be able to penetrate into biofilms (Anderl et al., 2000; Araujo et al., 2014), but the active concentrations achieved in the different layers of our biofilms are unknown. However, a statistically significant decrease in the total log_10_ cfu was observed for BIO CIP compared to BIO CTR during both experiments. This may indicate that the high levels of CIP treatment was a stressful challenge for the bacteria, even under the protection offered by the biofilm lifestyle. CIP is known to induce an SOS-response leading to mutagenesis (Qin et al., 2015). Our study was not designed to measure mutations rates. Still, crude observations of the total number of mutations observed in selected isolates may indicate a higher number of mutations in BIO CIP than in BIO CTR.

In the BIO CTR, isolates from four out of six parallel biofilms displayed different non-synonymous mutations in *fimH2*, and three had identical mutations in *pgm* (Gly494Val). This might indicate a selective advantage in long term biofilms for these mutants, as both these genes have been reported to be involved in biofilm formation. The FimH protein, which is the receptor-recognizing part of type 1 fimbriae (Krogfelt et al., 1990), has been shown to be important for biofilm formation by *E. coli* K-12 under static growth conditions (Pratt and Kolter, 1998), i.e., the same conditions as in our experiments. Furthermore, studies using *E. coli* transposon mutants showed that *pgm*, encoding phosphoglucomutase, influenced motility and curli production (Hadjifrangiskou et al., 2012), both factors that contribute to biofilm formation by *E. coli* (Nesse et al., 2021). Although we did not observe increased numbers of cfu in our control biofilms, these mutations might still be beneficious in other ways, e.g., by contributing to better adhesion.

Our experiments with planktonic bacteria were originally included as comparison for the biofilm experiments. However, interesting differences in the evolutionary trajectory of the two planktonic experiments were also observed. In experiment 2, the evolution followed the commonly reported trajectory, i.e., early observations of reduced susceptibility, and rapid mutations in the *gyrA* followed by mutations in genes encoding multidrug efflux-pump systems. In experiment 1, however, no growth on the selective agar plates was observed at the first three samplings. In addition, the total log_10_ cfu in the biofilm fell dramatically at sampling 3 where the CIP concentration was twice the MIC value of the wildtype strain. This indicates that no or only a very low level of reduced susceptibility had developed at this stage. The total log_10_ cfu was almost restored at sampling 4 where it included colonies growing on low and medium selective plates. Isolates from these plates harbored the same mutation in *gyrB* as observed in BIO CIP isolates, in addition to various mutations in multidrug efflux pump system genes. Colonies with mutation in *gyrA* were not observed until the last two samplings. The *gyrB* mutation can augment resistance if *gyrA* is already mutated, but it is less effective in a bacterial cell with wild-type *gyrA* (Jacoby, 2005; Bhatnagar and Wong, 2019). A small reduction in susceptibility due to the *gyrB* mutation observed may thus have given sufficient protection under the early exposures to sub-inhibitory and low concentrations. If so, this might have reduced the competitive advantage of other mutations at these concentrations. Furthermore, all isolates investigated displayed either an identical frameshift variant in the gene *rfbC* (4 isolates) or a conservative inframe insertion in *rfbD* (1 isolate). These genes belong to the *rfb* cluster encoding enzymes involved in O-antigen synthesis (Coimbra et al., 2000). The gene *rfbC* has also been reported to be involved in the response of *E. coli* to lethal stress, e.g., from exposure to the quinolone nalidixic acid (Han et al., 2010). It is therefore possible the mutations in *rfbC*, and maybe also in *rfbB*, may have contributed to reduced susceptibility to CIP.

In the PLANK CTR, three isolates from different samplings displayed identical mutations in the genes *fimH2*, *flu1*, and *yohF*. The two first genes encode surface structures involved in adhesion and agglutination (Krogfelt et al., 1990; Hasman et al., 1999), whereas the last one is a putative oxidoreductase. In contrast to the biofilm experiments, the planktonic samples were not taken from separately evolved cultures, but from cultures that were propagated from the preceding one. Whether the presence of these mutations over time in fact reflects a selective advantage is therefore uncertain.

Resistance to CIP evolved more rapidly in biofilm-residing bacteria than in planktonic ones. Colonies growing on selective plates with high CIP concentration were first observed at sampling 2 from BIO CIP and at sampling 5 and 6 from PLANK CIP. However, as the biofilms were subjected to an initial CIP concentration eight times higher than the planktonic bacteria, the CIP concentration at sampling 2 in the biofilm experiments was the same as at sampling 5 in the planktonic. The fast development in the biofilm residing bacteria therefore appears primarily to be due to the biofilm lifestyle permitting susceptible bacteria being exposed to the higher CIP concentrations. This is probably also the reason for the biofilm residing bacteria displaying higher numbers of resistance-conferring mutations and higher MIC values than the planktonic. This may indicate that the risk of resistance development is higher during AM treatment of biofilm infections than non-biofilm infections.

## Conclusion

This study displayed evolution of high level CIP resistance in biofilm-residing *E. coli* subjected to lethal CIP concentrations for up to 2 weeks. This is the first study showing evolution of AMR in bacteria continuously exposed to high AM concentrations while located within a biofilm. The resistance that evolved in our study was target based, i.e., conferred by mutations in topoisomerase genes. This is in contrast to earlier studies using short-term biofilms exposed to sub-inhibitory concentrations of CIP, where the resistance observed was low level and non-target based. Furthermore, we found that the resistance evolved more rapidly within the biofilm than in a planktonic suspension. This was most probably because the protection provided by the biofilm lifestyle allowed the susceptible bacteria to survive exposure to CIP concentrations above their MIC value. Biofilms are present and part of the pathogenesis in a number of bacterial infections, which are often treated with AM. Therefore, this study was performed at body temperature. Our findings indicate that AM treatment of biofilm-associated infections may pose a high risk of stimulating AMR development rather than eradication of the pathogens.

## Data availability statement

The datasets presented in this study can be found in online repositories. The names of the repository/repositories and accession number(s) can be found below: https://www.ebi.ac.uk/ena, PRJEB63533.

## Author contributions

SM: project administration and funding acquisition. BA, AO, and SM: experiments. LN and SM: data analyses. LN, AO, and SM: writing—original draft. All authors have conceptualization, writing—review and editing, read, and agreed to the submitted version of the manuscript.

## Footnotes

1
http://www.eucast.org

2
https://www.bioinformatics.babraham.ac.uk/projects/fastqc/

3
https://github.com/FelixKrueger/TrimGalore

4
https://github.com/tseemann/snippy
