# Supplementary material for: Evolution of antimicrobial resistance in E. coli biofilm treated with high doses of ciprofloxacin
Source: Front Microbiol. 2023 Sep 5;14:1246895. doi: 10.3389/fmicb.2023.1246895 (PMC10509014; doi:10.3389/fmicb.2023.1246895)
Supplement: Supplementary file 1 [file Data_Sheet_1.PDF]

Supplementary Material

Table S1. Results from MIC testing and WGS of selected isolates from BIO CTR experiment 2. MIC values are given in mg/L. CHL = chloramphenicol, TET = tetracycline. Only mutations in annotated genes are shown. For details on all mutations, see Supplementary file 2.

	**BIOFILM CONTROL**					
**Sampling no.**	**0**	**1**	**2**	**4**	**6**	**7**
Strain no.	02	03	04	05	06	07
**MIC CIP**	**≤0.015**	**≤0.015**	**≤0.015**	**≤0.015**	**0.03**	**0.03**
MIC CHL	≤ 8	≤ 8	≤ 8	≤ 8	≤ 8	≤ 8
MIC TET	≤ 2	≤ 2	≤ 2	≤ 2	4	4
**Total no. mutations**	**0**	**1**	**2**	**5**	**2**	**3**
clpX			Ala369Pro			
rpoS				stop gained		
rfbC						Arg23Leu
rfbD					stop gained	
fimH_2		Gly14-Asn18 deletion		Arg187Leu	Gly94Trp	Ala148Asp
pgm			Gly494Val	Gly494Val		Gly494Val
ydiV				Asn64Lys		
yohF				frameshift		

Table S2. Results from MIC testing and WGS of selected isolates from PLANK CTR experiment 2. MIC values are given in mg/L. CHL = chloramphenicol, TET = tetracycline. Only mutations in annotated genes are shown. For details on all mutations, see Supplementary file 4.

	**PLANKTONIC CONTROL**					
**Sampling no.**	**0**	**1**	**2**	**4**	**6**	**7**
Strain no.	08	09	10	11	12	13
**MIC CIP**	**≤0.015**	**≤0.015**	**≤0.015**	**≤0.015**	**0.03**	**≤0.015**
MIC CHL	≤ 8	≤ 8	≤ 8	≤ 8	≤ 8	≤ 8
MIC TET	≤ 2	≤ 2	≤ 2	4	4	≤ 2
**Total no. mutations**	**0**	**3**	**5**	**6**	**6**	**2**
rRNA		intergenic region				
rfbB					frameshift	
fimH2			Asp183Tyr	Asp183Tyr	Asp183Tyr	
agaS		His332His				
cytR						Arg334Leu
exoX					Ser185Cys	
Flu1			Asn537Lys	Asn537Lys	Asn537Lys	
por2						Ala88Val
yohF			Phe27Val	Phe27Val	Phe27Val	
