# Supplementary material for: Evolution of antimicrobial resistance in E. coli biofilm treated with high doses of ciprofloxacin
Source: Front Microbiol. 2023 Sep 5;14:1246895. doi: 10.3389/fmicb.2023.1246895 (PMC10509014; doi:10.3389/fmicb.2023.1246895)
Supplement: Supplementary file 2 [file Data_Sheet_2.PDF]

STRAIN 2

CHROM	POS	TYPE	REF	ALT	EVIDENCE	FTYPE	STRAND	NT_POS	AA_POS	EFFECT	LOCUS_TAG	GENE	PRODUCT

0 variants

STRAIN 3

CHROM	POS	TYPE	REF	ALT	EVIDENCE	FTYPE	STRAND	NT_POS	AA_POS
9	100000	del	GGGCTGGTCGGTAAAT	G	G:44 GGGCTGGTCGGTAAAT:0	CDS	+	41/903	14/300

EFFECT	LOCUS_TAG	GENE	PRODUCT
disruptive_inframe_deletion c.41_55delGCTGGTCGGTAAATG p.Gly14_Asn18del	IDEKCFHF_02378	fimH_2	Type 1 fimbrin D-mannose specific adhesin

STRAIN 4

CHROM	POS	TYPE	REF	ALT	EVIDENCE	FTYPE	STRAND	NT_POS	AA_POS	EFFECT	LOCUS_TAG	GENE
10	117405	snp	C	G	G:26 C:0	CDS	-	1105/1275	369/424	missense_variant c.1105G>C p.Ala369Pro	IDEKCFHF_02588	clpX
14	122055	snp	G	T	T:40 G:0	CDS	+	1481/1641	494/546	missense_variant c.1481G>T p.Gly494Val	IDEKCFHF_03202	pgm

PRODUCT
ATP-dependent Clp protease ATP-binding subunit ClpX
Phosphoglucomutase

STRAIN 5

CHROM	POS	TYPE	REF	ALT	EVIDENCE	FTYPE	STRAND	NT_POS	AA_POS	EFFECT	LOCUS_TAG	GENE
2	82723	snp	G	A	A:32 G:0	CDS	-	751/993	251/330	stop_gained c.751C>T p.Gln251*	IDEKCFHF_00655	rpoS
8	154341	snp	C	A	A:46 C:0	CDS	+	192/930	64/309	missense_variant c.192C>A p.Asn64Lys	IDEKCFHF_02240	tus
9	100521	snp	G	T	T:48 G:0	CDS	+	560/903	187/300	missense_variant c.560G>T p.Arg187Leu	IDEKCFHF_02378	fimH_2
14	122055	snp	G	T	T:42 G:0	CDS	+	1481/1641	494/546	missense_variant c.1481G>T p.Gly494Val	IDEKCFHF_03202	pgm
25	54765	del	CA	C	C:30 CA:0	CDS	-	14/714	5/237	frameshift_variant c.14delT p.Leu5fs	IDEKCFHF_04239	ydiV

PRODUCT
RNA polymerase sigma factor RpoS
DNA replication terminus site-binding protein
Type 1 fimbrin D-mannose specific adhesin
Phosphoglucomutase
Putative anti-FlhC(2)FlhD(4) factor YdiV

STRAIN 6

CHROM	POS	TYPE	REF	ALT	EVIDENCE	FTYPE	STRAND	NT_POS	AA_POS	EFFECT	LOCUS_TAG	GENE
6	57541	snp	C	G	G:45 C:0	CDS	+	215/900	72/299	stop_gained c.215C>G p.Ser72*	IDEKCFHF_01716	rfbD
9	100241	snp	G	T	T:50 G:0	CDS	+	280/903	94/300	missense_variant c.280G>T p.Gly94Trp	IDEKCFHF_02378	fimH_2
34	15596	snp	A	G	G:60 A:0							

PRODUCT
dTDP-4-dehydrorhamnose reductase
Type 1 fimbrin D-mannose specific adhesin

STRAIN 7

CHROM	POS	TYPE	REF	ALT	EVIDENCE	FTYPE	STRAND	NT_POS	AA_POS	EFFECT	LOCUS_TAG	GENE
6	59234	snp	G	T	T:32 G:0	CDS	+	68/552	23/183	missense_variant c.68G>T p.Arg23Leu	IDEKCFHF_01718	rfbC
9	100404	snp	C	A	A:31 C:0	CDS	+	443/903	148/300	missense_variant c.443C>A p.Ala148Asp	IDEKCFHF_02378	fimH_2
14	122055	snp	G	T	T:55 G:0	CDS	+	1481/1641	494/546	missense_variant c.1481G>T p.Gly494Val	IDEKCFHF_03202	pgm

PRODUCT
dTDP-4-dehydrorhamnose 3,5-epimerase
Type 1 fimbrin D-mannose specific adhesin
Phosphoglucomutase
