# Supplementary material for: Evolution of antimicrobial resistance in E. coli biofilm treated with high doses of ciprofloxacin
Source: Front Microbiol. 2023 Sep 5;14:1246895. doi: 10.3389/fmicb.2023.1246895 (PMC10509014; doi:10.3389/fmicb.2023.1246895)
Supplement: Supplementary file 3 [file Data_Sheet_3.PDF]

STRAIN 30

CHROM	POS	TYPE	REF	ALT	EVIDENCE	FTYPE	STRAND	NT_POS	AA_POS	EFFECT
1	237118	snp	G	A	A:36 G:0	CDS	-	248/2628	83/875	missense_variant c.248C>T p.Ser83Leu
6	131889	snp	T	G	G:54 T:0	CDS	+	186/1053	62/350	synonymous_variant c.186T>G p.Val62Val
7	117967	snp	G	T	T:38 G:0	CDS	-	240/2259	80/752	missense_variant c.240C>A p.Ser80Arg
13	100446	snp	A	G	G:48 A:0					
15	59761	del	TA	T	T:39 TA:0	CDS	-	133/1365	45/454	frameshift_variant c.133delT p.Tyr45fs
46	4875	complex	GGAGGCCA	TGGCCTCC	TGGCCTCC:36 GGAGGCCA:0					

LOCUS_TAG	GENE	PRODUCT
IDEKCFHF_00236	gyrA	DNA gyrase subunit A
IDEKCFHF_01792		hypothetical protein
IDEKCFHF_02007	parC	DNA topoisomerase 4 subunit A
IDEKCFHF_03264	mnmE	tRNA modification GTPase MnmE

STRAIN 31

CHROM	POS	TYPE	REF	ALT	EVIDENCE	FTYPE	STRAND
1	237118	snp	G	A	A:25 G:0	CDS	-
6	131889	snp	T	G	G:46 T:0	CDS	+
7	117967	snp	G	T	T:19 G:0	CDS	-
8	103181	ins	T	TGGGCAATATTATCCCCTGCAACTAATTACTTGCCA	TGGGCAATATTATCCCCTGCAACTAATTACTTGCCA:13 T:0		
13	100446	snp	A	G	G:27 A:0		

NT_POS	AA_POS	EFFECT	LOCUS_TAG	GENE	PRODUCT
248/2628	83/875	missense_variant c.248C>T p.Ser83Leu	IDEKCFHF_00236	gyrA	DNA gyrase subunit A
186/1053	62/350	synonymous_variant c.186T>G p.Val62Val	IDEKCFHF_01792		hypothetical protein
240/2259	80/752	missense_variant c.240C>A p.Ser80Arg	IDEKCFHF_02007	parC	DNA topoisomerase 4 subunit A

STRAIN 32

CHROM	POS	TYPE	REF	ALT	EVIDENCE	FTYPE	STRAND	NT_POS	AA_POS
1	237107	snp	C	T	T:35 C:0	CDS	-	259/2628	87/875
8	103537	snp	G	T	T:37 G:0	CDS	+	310/435	104/144
10	90142	del	TTTC	T	T:47 TTTC:0	CDS	-	33/648	11/215
12	46864	del	GCGTCT	G	G:32 GCGTCT:0	CDS	+	622/639	208/212
15	17761	del	TCGCGTATTTCTGG	T	T:28 TCGCGTATTTCTGG:0	CDS	+	1807/1890	603/629
20	55291	del	AACCTGCACCTGCACCTGC	A	A:14 AACCTGCACCTGCACCTGC:0	CDS	+	1197/1254	399/417
24	49278	snp	G	T	T:41 G:0	CDS	-	183/1389	61/462

EFFECT	LOCUS_TAG	GENE
missense_variant c.259G>A p.Asp87Asn	IDEKCFHF_00236	gyrA
missense_variant c.310G>T p.Gly104Cys	IDEKCFHF_02189	marR
conservative_inframe_deletion c.31_33delGAA p.Glu11del	IDEKCFHF_02563	acrR_1
frameshift_variant c.622_626delCGTCT p.Arg208fs	IDEKCFHF_02825	sspA
frameshift_variant c.1807_1819delCGTATTTCTGGCG p.Arg603fs	IDEKCFHF_03226	mnmG
disruptive_inframe_deletion c.1197_1214delTGCACCTGCACCTGCACC p.Ala400_Pro405del	IDEKCFHF_03818	hemX
missense_variant c.183C>A p.Phe61Leu	IDEKCFHF_04158	dnaB_2

PRODUCT
DNA gyrase subunit A
Multiple antibiotic resistance protein MarR
HTH-type transcriptional regulator AcrR
Stringent starvation protein A
tRNA uridine 5-carboxymethylaminomethyl modification enzyme MnmG
Protein HemX
Replicative DNA helicase

STRAIN 33

CHROM	POS	TYPE	REF	ALT	EVIDENCE	FTYPE	STRAND	NT_POS	AA_POS	EFFECT	LOCUS_TAG	GENE
1	96633	snp	G	T	T:34 G:0							
1	237106	snp	T	C	C:21 T:0	CDS	-	260/2628	87/875	missense_variant c.260A>G p.Asp87Gly	IDEKCFHF_00236	gyrA
4	73574	snp	G	T	T:42 G:0	CDS	-	232/537	78/178	missense_variant c.232C>A p.Arg78Ser	IDEKCFHF_01318	hpt
4	222199	snp	A	T	T:46 A:0	CDS	+	581/870	194/289	missense_variant c.581A>T p.Gln194Leu	IDEKCFHF_01443	rob_1
6	58347	ins	A	ATGGC	ATGGC:25 A:2	CDS	+	71/879	24/292	frameshift_variant c.67_70dupGCTG p.Val24fs	IDEKCFHF_01717	rfbA
7	124028	snp	A	G	G:18 A:0	CDS	-	1372/1893	458/630	missense_variant c.1372T>C p.Ser458Pro	IDEKCFHF_02016	parE
10	89569	ins	G	GATAC	GATAC:8 G:0	CDS	-	606/648	202/215	frameshift_variant c.603_606dupGTAT p.Leu203fs	IDEKCFHF_02563	acrR_1
10	117467	snp	A	T	T:11 A:0	CDS	-	1043/1275	348/424	missense_variant c.1043T>A p.Leu348Gln	IDEKCFHF_02588	clpX
12	122	snp	T	C	C:30 T:2							
15	67993	snp	C	A	A:57 C:0	CDS	+	1391/2415	464/804	missense_variant c.1391C>A p.Ser464Tyr	IDEKCFHF_03271	gyrB

PRODUCT

DNA gyrase subunit A
Hypoxanthine phosphoribosyltransferase
Right origin-binding protein
Glucose-1-phosphate thymidylyltransferase 1
DNA topoisomerase 4 subunit B
HTH-type transcriptional regulator AcrR
ATP-dependent Clp protease ATP-binding subunit ClpX

DNA gyrase subunit B

STRAIN 34

CHROM	POS	TYPE	REF	ALT	EVIDENCE	FTYPE	STRAND	NT_POS	AA_POS	EFFECT
1	237106	snp	T	C	C:28 T:0	CDS	-	260/2628	87/875	missense_variant c.260A>G p.Asp87Gly
8	103426	snp	G	T	T:32 G:0	CDS	+	199/435	67/144	missense_variant c.199G>T p.Asp67Tyr
10	89944	snp	G	T	T:39 G:0	CDS	-	232/648	78/215	missense_variant c.232C>A p.Gln78Lys
12	140331	ins	T	TTGTTGAA	TTGTTGAA:29 T:2	CDS	-	272/336	91/111	frameshift_variant c.266_272dupTTCAACA p.Gln91fs
16	33081	snp	T	C	C:31 T:0	CDS	+	1851/2634	617/877	synonymous_variant c.1851T>C p.Leu617Leu

LOCUS_TAG	GENE	PRODUCT
IDEKCFHF_00236	gyrA	DNA gyrase subunit A
IDEKCFHF_02189	marR	Multiple antibiotic resistance protein MarR
IDEKCFHF_02563	acrR_1	HTH-type transcriptional regulator AcrR
IDEKCFHF_02922	prlF	Antitoxin PrlF
IDEKCFHF_03365	mngB	Mannosylglycerate hydrolase

STRAIN 35

CHROM	POS	TYPE	REF	ALT	EVIDENCE	FTYPE	STRAND	NT_POS	AA_POS	EFFECT	LOCUS_TAG	GENE
1	237106	snp	T	C	C:47 T:0	CDS	-	260/2628	87/875	missense_variant c.260A>G p.Asp87Gly	IDEKCFHF_00236	gyrA
8	103426	snp	G	T	T:28 G:0	CDS	+	199/435	67/144	missense_variant c.199G>T p.Asp67Tyr	IDEKCFHF_02189	marR
10	89944	snp	G	T	T:55 G:0	CDS	-	232/648	78/215	missense_variant c.232C>A p.Gln78Lys	IDEKCFHF_02563	acrR_1
16	33081	snp	T	C	C:34 T:0	CDS	+	1851/2634	617/877	synonymous_variant c.1851T>C p.Leu617Leu	IDEKCFHF_03365	mngB

PRODUCT
DNA gyrase subunit A
Multiple antibiotic resistance protein MarR
HTH-type transcriptional regulator AcrR
Mannosylglycerate hydrolase

STRAIN 36

CHROM	POS	TYPE	REF	ALT	EVIDENCE	FTYPE	STRAND	NT_POS	AA_POS	EFFECT
1	237107	snp	C	T	T:36 C:0	CDS	-	259/2628	87/875	missense_variant c.259G>A p.Asp87Asn
1	243237	snp	C	G	G:39 C:0	CDS	+	489/2286	163/761	synonymous_variant c.489C>G p.Gly163Gly
3	46669	ins	C	CGCTGGCG	CGCTGGCG:25 C:0	CDS	-	1619/3186	540/1061	frameshift_variant c.1613_1619dupCGCCAGC p.Pro541fs
7	117967	snp	G	T	T:27 G:0	CDS	-	240/2259	80/752	missense_variant c.240C>A p.Ser80Arg
15	67993	snp	C	A	A:33 C:0	CDS	+	1391/2415	464/804	missense_variant c.1391C>A p.Ser464Tyr
38	19556	snp	A	T	T:34 A:0	CDS	+	210/600	70/199	synonymous_variant c.210A>T p.Val70Val

LOCUS_TAG	GENE	PRODUCT
IDEKCFHF_00236	gyrA	DNA gyrase subunit A
IDEKCFHF_00239	nrdA	Ribonucleoside-diphosphate reductase 1 subunit alpha
IDEKCFHF_00981	rne	Ribonuclease E
IDEKCFHF_02007	parC	DNA topoisomerase 4 subunit A
IDEKCFHF_03271	gyrB	DNA gyrase subunit B
IDEKCFHF_04860		hypothetical protein

STRAIN 37

CHROM	POS	TYPE	REF	ALT	EVIDENCE	FTYPE	STRAND	NT_POS	AA_POS	EFFECT
1	237118	snp	G	A	A:57 G:0	CDS	-	248/2628	83/875	missense_variant c.248C>T p.Ser83Leu
3	155239	del	TCTGGTGGA	T	T:41 TCTGGTGGA:0	CDS	+	292/834	98/277	frameshift_variant c.292_299delCTGGTGGA p.Leu98fs
5	106566	snp	G	T	T:53 G:0	CDS	+	151/1089	51/362	stop_gained c.151G>T p.Glu51*
7	117967	snp	G	T	T:62 G:0	CDS	-	240/2259	80/752	missense_variant c.240C>A p.Ser80Arg
8	103440	del	CA	C	C:69 CA:0	CDS	+	214/435	72/144	frameshift_variant c.214delA p.Thr72fs
10	90138	snp	C	A	A:81 C:0	CDS	-	38/648	13/215	missense_variant c.38G>T p.Arg13Leu
14	122055	snp	G	T	T:52 G:0	CDS	+	1481/1641	494/546	missense_variant c.1481G>T p.Gly494Val
15	68000	snp	A	C	C:65 A:0	CDS	+	1398/2415	466/804	missense_variant c.1398A>C p.Glu466Asp

LOCUS_TAG	GENE	PRODUCT
IDEKCFHF_00236	gyrA	DNA gyrase subunit A
IDEKCFHF_01088	prmC	Release factor glutamine methyltransferase
IDEKCFHF_01564	ompF	Outer membrane porin F
IDEKCFHF_02007	parC	DNA topoisomerase 4 subunit A
IDEKCFHF_02189	marR	Multiple antibiotic resistance protein MarR
IDEKCFHF_02563	acrR_1	HTH-type transcriptional regulator AcrR
IDEKCFHF_03202	pgm	Phosphoglucomutase
IDEKCFHF_03271	gyrB	DNA gyrase subunit B
