# Supplementary material for: Evolution of antimicrobial resistance in E. coli biofilm treated with high doses of ciprofloxacin
Source: Front Microbiol. 2023 Sep 5;14:1246895. doi: 10.3389/fmicb.2023.1246895 (PMC10509014; doi:10.3389/fmicb.2023.1246895)
Supplement: Supplementary file 4 [file Data_Sheet_4.PDF]

STRAIN 8

CHROM	POS	TYPE	REF	ALT	EVIDENCE	FTYPE	STRAND	NT_POS	AA_POS	EFFECT	LOCUS_TAG	GENE	PRODUCT

0 variants

STRAIN 9

CHROM	POS	TYPE	REF	ALT	EVIDENCE	FTYPE	STRAND	NT_POS	AA_POS	EFFECT	LOCUS_TAG	GENE
12	122	snp	T	C	C:35 T:1							
12	131	snp	A	C	C:28 A:1	rRNA	+			intergenic_region n.131A>C	IDEKCFHF_02783	
12	132329	snp	G	A	A:49 G:0	CDS	-	996/1155	332/384	synonymous_variant c.996C>T p.His332His	IDEKCFHF_02913	agaS

PRODUCT

5S ribosomal RNA
Putative D-galactosamine-6-phosphate deaminase AgaS

STRAIN 10

CHROM	POS	TYPE	REF	ALT	EVIDENCE	FTYPE	STRAND	NT_POS	AA_POS	EFFECT	LOCUS_TAG
1	92068	snp	A	C	C:37 A:0	CDS	-	79/762	27/253	missense_variant c.79T>G p.Phe27Val	IDEKCFHF_00092
6	208486	snp	C	T	T:41 C:0						
9	100508	snp	G	T	T:25 G:0	CDS	+	547/903	183/300	missense_variant c.547G>T p.Asp183Tyr	IDEKCFHF_02378
11	158594	snp	A	C	C:37 A:0	CDS	-	1611/1707	537/568	missense_variant c.1611T>G p.Asn537Lys	IDEKCFHF_02781
34	14424	del	GAAGATAAC	G	G:40 GAAGATAAC:0						

GENE	PRODUCT
yohF	putative oxidoreductase YohF
fimH_2	Type 1 fimbrin D-mannose specific adhesin
flu_1	Antigen 43

STRAIN 11

CHROM	POS	TYPE	REF	ALT	EVIDENCE	FTYPE	STRAND	NT_POS	AA_POS	EFFECT
1	92068	snp	A	C	C:46 A:0	CDS	-	79/762	27/253	missense_variant c.79T>G p.Phe27Val
6	65801	snp	G	T	T:43 G:0	CDS	+	588/885	196/294	missense_variant c.588G>T p.Trp196Cys
6	208486	snp	C	T	T:45 C:0					
9	100508	snp	G	T	T:36 G:0	CDS	+	547/903	183/300	missense_variant c.547G>T p.Asp183Tyr
11	158594	snp	A	C	C:64 A:0	CDS	-	1611/1707	537/568	missense_variant c.1611T>G p.Asn537Lys
46	4875	complex	GGAGGCCA	TGGCCTCC	TGGCCTCC:26 GGAGGCCA:0					

LOCUS_TAG	GENE	PRODUCT
IDEKCFHF_00092	yohF	putative oxidoreductase YohF
IDEKCFHF_01724		hypothetical protein
IDEKCFHF_02378	fimH_2	Type 1 fimbrin D-mannose specific adhesin
IDEKCFHF_02781	flu_1	Antigen 43

STRAIN 12

CHROM	POS	TYPE	REF	ALT	EVIDENCE	FTYPE	STRAND	NT_POS	AA_POS	EFFECT	LOCUS_TAG	GENE
1	92068	snp	A	C	C:41 A:0	CDS	-	79/762	27/253	missense_variant c.79T>G p.Phe27Val	IDEKCFHF_00092	yohF
6	56352	ins	C	CA	CA:40 C:0	CDS	+	112/1086	38/361	frameshift_variant c.111_112insA p.Gly38fs	IDEKCFHF_01715	rfbB
6	208486	snp	C	T	T:36 C:0							
9	100508	snp	G	T	T:36 G:0	CDS	+	547/903	183/300	missense_variant c.547G>T p.Asp183Tyr	IDEKCFHF_02378	fimH_2
11	158594	snp	A	C	C:32 A:0	CDS	-	1611/1707	537/568	missense_variant c.1611T>G p.Asn537Lys	IDEKCFHF_02781	flu_1
27	47461	snp	C	G	G:20 C:0	CDS	+	554/663	185/220	missense_variant c.554C>G p.Ser185Cys	IDEKCFHF_04392	exoX

PRODUCT
putative oxidoreductase YohF
dTDP-glucose 4,6-dehydratase

Type 1 fimbrin D-mannose specific adhesin
Antigen 43
Exodeoxyribonuclease 10

STRAIN 13

CHROM	POS	TYPE	REF	ALT	EVIDENCE	FTYPE	STRAND	NT_POS	AA_POS	EFFECT	LOCUS_TAG	GENE
8	121010	snp	G	A	A:35 G:0	CDS	-	263/1461	88/486	missense_variant c.263C>T p.Ala88Val	IDEKCFHF_02208	por_2
22	4013	snp	G	T	T:40 G:0	CDS	+	1001/1026	334/341	missense_variant c.1001G>T p.Arg334Leu	IDEKCFHF_03940	cytR

PRODUCT
Polyol:NADP oxidoreductase
HTH-type transcriptional repressor CytR
