# Supplementary material for: Evolution of antimicrobial resistance in E. coli biofilm treated with high doses of ciprofloxacin
Source: Front Microbiol. 2023 Sep 5;14:1246895. doi: 10.3389/fmicb.2023.1246895 (PMC10509014; doi:10.3389/fmicb.2023.1246895)
Supplement: Supplementary file 5 [file Data_Sheet_5.PDF]

STRAIN 14

CHROM	POS	TYPE	REF	ALT	EVIDENCE	FTYPE	STRAND	NT_POS	AA_POS	EFFECT	LOCUS_TAG	GENE
1	237118	snp	G	A	A:18 G:0	CDS	-	248/2628	83/875	missense_variant c.248C>T p.Ser83Leu	IDEKCFHF_00236	gyrA
3	96044	snp	T	G	G:36 T:0	CDS	-	635/642	212/213	missense_variant c.635A>C p.Glu212Ala	IDEKCFHF_01028	hflD
12	79217	snp	C	T	T:26 C:0	CDS	+	147/294	49/97	synonymous_variant c.147C>T p.Arg49Arg	IDEKCFHF_02858	mlaB

PRODUCT
DNA gyrase subunit A
High frequency lysogenization protein HflD
Intermembrane phospholipid transport system binding protein MlaB

STRAIN 15

CHROM	POS	TYPE	REF	ALT	EVIDENCE	FTYPE	STRAND	NT_POS	AA_POS	EFFECT	LOCUS_TAG	GENE
1	237118	snp	G	A	A:35 G:0	CDS	-	248/2628	83/875	missense_variant c.248C>T p.Ser83Leu	IDEKCFHF_00236	gyrA
8	103508	snp	G	A	A:26 G:0	CDS	+	281/435	94/144	missense_variant c.281G>A p.Arg94His	IDEKCFHF_02189	marR
14	118211	snp	T	A	A:42 T:0	CDS	-	272/531	91/176	missense_variant c.272A>T p.Gln91Leu	IDEKCFHF_03198	fldA

PRODUCT
DNA gyrase subunit A
Multiple antibiotic resistance protein MarR
Flavodoxin 1

STRAIN 16

CHROM	POS	TYPE	REF	ALT	EVIDENCE	FTYPE	STRAND	NT_POS	AA_POS	EFFECT
1	237118	snp	G	A	A:24 G:0	CDS	-	248/2628	83/875	missense_variant c.248C>T p.Ser83Leu
8	103435	del	GCACTCAC	G	G:28 GCACTCAC:0	CDS	+	210/435	70/144	frameshift_variant c.210_216delACTCACC p.Leu71fs

LOCUS_TAG	GENE	PRODUCT
IDEKCFHF_00236	gyrA	DNA gyrase subunit A
IDEKCFHF_02189	marR	Multiple antibiotic resistance protein MarR

STRAIN 17

CHROM	POS	TYPE	REF	ALT	EVIDENCE	FTYPE	STRAND	NT_POS	AA_POS	EFFECT	LOCUS_TAG	GENE	PRODUCT
1	237118	snp	G	A	A:22 G:0	CDS	-	248/2628	83/875	missense_variant c.248C>T p.Ser83Leu	IDEKCFHF_00236	gyrA	DNA gyrase subunit A

STRAIN 18

CHROM	POS	TYPE	REF	ALT	EVIDENCE	FTYPE	STRAND	NT_POS	AA_POS	EFFECT	LOCUS_TAG	GENE	PRODUCT
1	237118	snp	G	A	A:33 G:0	CDS	-	248/2628	83/875	missense_variant c.248C>T p.Ser83Leu	IDEKCFHF_00236	gyrA	DNA gyrase subunit A

STRAIN 19

CHROM	POS	TYPE	REF	ALT	EVIDENCE	FTYPE	STRAND	NT_POS	AA_POS	EFFECT	LOCUS_TAG	GENE
1	237118	snp	G	A	A:31 G:0	CDS	-	248/2628	83/875	missense_variant c.248C>T p.Ser83Leu	IDEKCFHF_00236	gyrA
8	103508	snp	G	A	A:38 G:0	CDS	+	281/435	94/144	missense_variant c.281G>A p.Arg94His	IDEKCFHF_02189	marR

PRODUCT
DNA gyrase subunit A
Multiple antibiotic resistance protein MarR

STRAIN 20

CHROM	POS	TYPE	REF	ALT	EVIDENCE	FTYPE	STRAND	NT_POS	AA_POS	EFFECT	LOCUS_TAG	GENE
1	237118	snp	G	A	A:24 G:0	CDS	-	248/2628	83/875	missense_variant c.248C>T p.Ser83Leu	IDEKCFHF_00236	gyrA
3	46435	ins	G	GTC	GTC:24 G:0	CDS	-	1853/3186	618/1061	frameshift_variant c.1852_1853dupGA p.Asp618fs	IDEKCFHF_00981	rne
6	59536	del	CTGAG	C	C:33 CTGAG:0	CDS	+	375/552	125/183	frameshift_variant c.375_378delTGAG p.Ser125fs	IDEKCFHF_01718	rfbC

PRODUCT
DNA gyrase subunit A
Ribonuclease E
dTDP-4-dehydrorhamnose 3,5-epimerase

STRAIN 21

CHROM	POS	TYPE	REF	ALT	EVIDENCE	FTYPE	STRAND	NT_POS	AA_POS
1	237118	snp	G	A	A:28 G:0	CDS	-	248/2628	83/875
4	205897	del	GGATGCCTTTATCCT	G	G:32 GGATGCCTTTATCCT:0	CDS	-	75/588	21/195
6	65607	snp	A	T	T:35 A:0	CDS	+	394/885	132/294
6	208488	snp	G	A	A:46 G:0				
7	167520	snp	G	A	A:44 G:0	CDS	-	191/1521	64/506
15	59137	snp	T	C	C:34 T:0	CDS	-	758/1365	253/454
34	14424	ins	G	GAAGATAAC	GAAGATAAC:39 G:2				

EFFECT	LOCUS_TAG	GENE	PRODUCT
missense_variant c.248C>T p.Ser83Leu	IDEKCFHF_00236	gyrA	DNA gyrase subunit A
frameshift_variant c.62_75delAGGATAAAGGCATC p.Gln21fs	IDEKCFHF_01429	mog	Molybdopterin adenylyltransferase
missense_variant c.394A>T p.Ile132Phe	IDEKCFHF_01724		hypothetical protein
missense_variant c.191C>T p.Ala64Val	IDEKCFHF_02056	aer	Aerotaxis receptor
missense_variant c.758A>G p.Asp253Gly	IDEKCFHF_03264	mnmE	tRNA modification GTPase MnmE

STRAIN 22

CHROM	POS	TYPE	REF	ALT	EVIDENCE	FTYPE	STRAND	NT_POS	AA_POS
1	237118	snp	G	A	A:29 G:0	CDS	-	248/2628	83/875
9	100328	del	CCGTGGCCGGTGG	C	C:23 CCGTGGCCGGTGG:0	CDS	+	370/903	124/300
11	68164	snp	G	C	C:30 G:0	CDS	+	380/465	127/154
24	51506	del	CG	C	C:13 CG:0				
24	51524	snp	C	A	A:13 C:0				

EFFECT	LOCUS_TAG	GENE	PRODUCT
missense_variant c.248C>T p.Ser83Leu	IDEKCFHF_00236	gyrA	DNA gyrase subunit A
conservative_inframe_deletion c.370_381delTGGCCGGTGGCG p.Trp124_Ala127del	IDEKCFHF_02378	fimH_2	Type 1 fimbrin D-mannose specific adhesin
missense_variant c.380G>C p.Arg127Pro	IDEKCFHF_02695	soxR	Redox-sensitive transcriptional activator SoxR

STRAIN 23

CHROM	POS	TYPE	REF	ALT	EVIDENCE	FTYPE	STRAND	NT_POS	AA_POS
1	237118	snp	G	A	A:30 G:0	CDS	-	248/2628	83/875
1	561758	snp	G	A	A:28 G:0	CDS	-	2730/4044	910/1347
6	65893	del	TA	T	T:40 TA:0	CDS	+	688/885	230/294
8	103618	ins	G	GAAGTGGC	GAAGTGGC:28 G:1	CDS	+	401/435	134/144
9	100336	del	GGTGGCGCTTTATTTGACGCCT	G	G:26 GGTGGCGCTTTATTTGACGCCT:0	CDS	+	379/903	127/300
16	37837	snp	G	A	A:38 G:0	CDS	+	276/294	92/97

EFFECT	LOCUS_TAG	GENE
missense_variant c.248C>T p.Ser83Leu	IDEKCFHF_00236	gyrA
synonymous_variant c.2730C>T p.Arg910Arg	IDEKCFHF_00536	purL
frameshift_variant c.688delA p.Ile230fs	IDEKCFHF_01724	
frameshift_variant c.394_400dupGTGGCAA p.Thr134fs	IDEKCFHF_02189	marR
conservative_inframe_deletion c.379_399delGCGCTTTATTTGACGCCTGTG p.Ala127_Val133del	IDEKCFHF_02378	fimH_2
synonymous_variant c.276G>A p.Leu92Leu	IDEKCFHF_03369	

PRODUCT
DNA gyrase subunit A
Phosphoribosylformylglycinamidine synthase
hypothetical protein
Multiple antibiotic resistance protein MarR
Type 1 fimbrin D-mannose specific adhesin
hypothetical protein

STRAIN 24

CHROM	POS	TYPE	REF	ALT	EVIDENCE	FTYPE	STRAND	NT_POS	AA_POS	EFFECT
1	237118	snp	G	A	A:34 G:0	CDS	-	248/2628	83/875	missense_variant c.248C>T p.Ser83Leu
6	65607	snp	A	T	T:48 A:0	CDS	+	394/885	132/294	missense_variant c.394A>T p.Ile132Phe
6	208488	snp	G	A	A:65 G:0					
7	167520	snp	G	A	A:54 G:0	CDS	-	191/1521	64/506	missense_variant c.191C>T p.Ala64Val
7	202077	snp	G	T	T:38 G:0	CDS	+	526/663	176/220	missense_variant c.526G>T p.Gly176Cys
11	68181	ins	C	CGTAA	CGTAA:33 C:0	CDS	+	402/465	134/154	frameshift_variant&stop_gained c.398_401dupGTAA p.Asn134fs

LOCUS_TAG	GENE	PRODUCT
IDEKCFHF_00236	gyrA	DNA gyrase subunit A
IDEKCFHF_01724		hypothetical protein
IDEKCFHF_02056	aer	Aerotaxis receptor
IDEKCFHF_02082	yqjA	Inner membrane protein YqjA
IDEKCFHF_02695	soxR	Redox-sensitive transcriptional activator SoxR

STRAIN 25

CHROM	POS	TYPE	REF	ALT	EVIDENCE	FTYPE	STRAND	NT_POS	AA_POS
6	59358	del	GT	G	G:41 GT:0	CDS	+	194/552	65/183
6	208488	snp	G	A	A:34 G:0				
8	103544	del	CAATATGCGAACAA	C	C:26 CAATATGCGAACAA:0	CDS	+	318/435	106/144
15	67993	snp	C	A	A:32 C:0	CDS	+	1391/2415	464/804

EFFECT	LOCUS_TAG	GENE	PRODUCT
frameshift_variant c.194delT p.Leu65fs	IDEKCFHF_01718	rfbC	dTDP-4-dehydrorhamnose 3,5-epimerase
frameshift_variant c.318_330delAATATGCGAACAA p.Ile107fs	IDEKCFHF_02189	marR	Multiple antibiotic resistance protein MarR
missense_variant c.1391C>A p.Ser464Tyr	IDEKCFHF_03271	gyrB	DNA gyrase subunit B

STRAIN 26

CHROM	POS	TYPE	REF	ALT	EVIDENCE	FTYPE	STRAND	NT_POS	AA_POS	EFFECT	LOCUS_TAG	GENE
1	483666	snp	A	G	G:31 A:0	CDS	+	322/849	108/282	missense_variant c.322A>G p.Thr108Ala	IDEKCFHF_00470	focB
6	59358	del	GT	G	G:47 GT:0	CDS	+	194/552	65/183	frameshift_variant c.194delT p.Leu65fs	IDEKCFHF_01718	rfbC
6	208488	snp	G	A	A:31 G:0							
10	89989	snp	A	G	G:56 A:0	CDS	-	187/648	63/215	missense_variant c.187T>C p.Trp63Arg	IDEKCFHF_02563	acrR_1
11	67936	snp	G	A	A:59 G:0	CDS	+	152/465	51/154	missense_variant c.152G>A p.Arg51His	IDEKCFHF_02695	soxR
15	67993	snp	C	A	A:51 C:0	CDS	+	1391/2415	464/804	missense_variant c.1391C>A p.Ser464Tyr	IDEKCFHF_03271	gyrB

PRODUCT
putative formate transporter 2
dTDP-4-dehydrorhamnose 3,5-epimerase

HTH-type transcriptional regulator AcrR
Redox-sensitive transcriptional activator SoxR
DNA gyrase subunit B

STRAIN 27

CHROM	POS	TYPE	REF	ALT	EVIDENCE	FTYPE	STRAND
3	97540	ins	C	CGA	CGA:29 C:0	CDS	-
6	57825	ins	T	TTGCGTC	TTGCGTC:36 T:0	CDS	+
6	208488	snp	G	A	A:41 G:0		
8	103549	ins	T	TGC	TGC:48 T:0	CDS	+
9	100216	del	TGGCGGCGTGTTATCTAATTTTTCCGGGACCGTAAAA	T	T:19 TGGCGGCGTGTTATCTAATTTTTCCGGGACCGTAAAA:0	CDS	+
10	90086	ins	T	TGATA	TGATA:34 T:0	CDS	-
12	46602	del	GTTCAGCTTCTGAA	G	G:31 GTTCAGCTTCTGAA:0	CDS	+
15	67990	del	CTTCTCAGGA	C	C:37 CTTCTCAGGA:0	CDS	+
20	57804	del	GCTGTTGCGGGGAAT	G	G:23 GCTGTTGCGGGGAAT:0	CDS	-
43	1576	ins	G	GTCT	GTCT:25 G:1	CDS	-

NT_POS	AA_POS	EFFECT	LOCUS_TAG	GENE
280/1107	94/368	frameshift_variant c.279_280dupTC p.Arg94fs	IDEKCFHF_01029	mnmA
508/900	170/299	conservative_inframe_insertion c.502_507dupCGTCTG p.Arg168_Leu169dup	IDEKCFHF_01716	rfbD
326/435	109/144	frameshift_variant c.324_325dupCG p.Glu109fs	IDEKCFHF_02189	marR
256/903	86/300	conservative_inframe_deletion c.256_291delGGCGGCGTGTTATCTAATTTTTCCGGGACCGTAAAA [illegible]	IDEKCFHF_02378	fimH_2
89/648	30/215	frameshift_variant c.86_89dupTATC p.Ser31fs	IDEKCFHF_02563	acrR_1
360/639	120/212	frameshift_variant c.360_372delTTCAGCTTCTGAA p.Ser121fs	IDEKCFHF_02825	sspA
1389/2415	463/804	disruptive_inframe_deletion c.1389_1397delTTCTCAGGA p.Ser464_Glu466del	IDEKCFHF_03271	gyrB
972/1236	320/411	frameshift_variant c.959_972delATTCCCCGCAACAG p.Asp320fs	IDEKCFHF_03821	chuR_2
167/612	56/203	disruptive_inframe_insertion c.165_167dupAGA p.Glu55dup	IDEKCFHF_04922	ruvA

PRODUCT
tRNA-specific 2-thiouridylase MnmA
dTDP-4-dehydrorhamnose reductase

Multiple antibiotic resistance protein MarR
Type 1 fimbrin D-mannose specific adhesin
HTH-type transcriptional regulator AcrR
Stringent starvation protein A
DNA gyrase subunit B
Anaerobic sulfatase-maturating enzyme
Holliday junction ATP-dependent DNA helicase RuvA

STRAIN 28

CHROM	POS	TYPE	REF	ALT	EVIDENCE	FTYPE	STRAND	NT_POS	AA_POS	EFFECT	LOCUS_TAG	GENE
1	237106	snp	T	C	C:34 T:0	CDS	-	260/2628	87/875	missense_variant c.260A>G p.Asp87Gly	IDEKCFHF_00236	gyrA
6	59358	del	GT	G	G:31 GT:0	CDS	+	194/552	65/183	frameshift_variant c.194delT p.Leu65fs	IDEKCFHF_01718	rfbC
6	208488	snp	G	A	A:47 G:0							
11	67936	snp	G	A	A:42 G:0	CDS	+	152/465	51/154	missense_variant c.152G>A p.Arg51His	IDEKCFHF_02695	soxR
15	67993	snp	C	A	A:40 C:0	CDS	+	1391/2415	464/804	missense_variant c.1391C>A p.Ser464Tyr	IDEKCFHF_03271	gyrB
35	5083	snp	C	T	T:41 C:0	CDS	-	279/705	93/234	missense_variant c.279G>A p.Met93Ile	IDEKCFHF_04761	fabR

PRODUCT
DNA gyrase subunit A
dTDP-4-dehydrorhamnose 3,5-epimerase

Redox-sensitive transcriptional activator SoxR
DNA gyrase subunit B
HTH-type transcriptional repressor FabR

STRAIN 29

CHROM	POS	TYPE	REF	ALT	EVIDENCE	FTYPE	STRAND	NT_POS	AA_POS	EFFECT	LOCUS_TAG	GENE
1	237106	snp	T	C	C:46 T:0	CDS	-	260/2628	87/875	missense_variant c.260A>G p.Asp87Gly	IDEKCFHF_00236	gyrA
3	136934	snp	C	T	T:45 C:0	CDS	-	1037/1419	346/472	stop_gained c.1037G>A p.Trp346*	IDEKCFHF_01073	dhaM
6	59358	del	GT	G	G:56 GT:0	CDS	+	194/552	65/183	frameshift_variant c.194delT p.Leu65fs	IDEKCFHF_01718	rfbC
6	208488	snp	G	A	A:52 G:0							
11	67936	snp	G	A	A:55 G:0	CDS	+	152/465	51/154	missense_variant c.152G>A p.Arg51His	IDEKCFHF_02695	soxR
15	67993	snp	C	A	A:35 C:0	CDS	+	1391/2415	464/804	missense_variant c.1391C>A p.Ser464Tyr	IDEKCFHF_03271	gyrB
18	90939	snp	A	C	C:33 A:0	CDS	+	79/990	27/329	missense_variant c.79A>C p.Thr27Pro	IDEKCFHF_03662	rpoA

PRODUCT
DNA gyrase subunit A
PEP-dependent dihydroxyacetone kinase, phosphoryl donor subunit DhaM
dTDP-4-dehydrorhamnose 3,5-epimerase

Redox-sensitive transcriptional activator SoxR
DNA gyrase subunit B
DNA-directed RNA polymerase subunit alpha
